# rRNA:mRNA pairing alters the length and the symmetry of mRNA-protected fragments in ribosome profiling experiments

**DOI:** 10.1093/bioinformatics/btt184

**Published:** 2013-04-19

**Authors:** Patrick B. F. O’Connor, Gene-Wei Li, Jonathan S. Weissman, John F. Atkins, Pavel V. Baranov

**Affiliations:** ^1^Department of Biochemistry, University College Cork, Cork, Ireland and ^2^Department of Cellular and Molecular Pharmacology, Howard Hughes Medical Institute, University of California, San Francisco, CA 94158, USA

## Abstract

**Motivation:** Ribosome profiling is a new technique that allows monitoring locations of translating ribosomes on mRNA at a whole transcriptome level. A recent ribosome profiling study demonstrated that internal Shine–Dalgarno (SD) sequences have a major global effect on translation rates in bacteria: ribosomes pause at SD sites in mRNA. Therefore, it is important to understand how SD sites effect mRNA movement through the ribosome and generation of ribosome footprints.

**Results:** Here, we provide evidence that in addition to pausing effect, internal SD sequences induce a caterpillar-like movement of mRNA through the ribosome cavity. Once an SD site binds to the ribosome, it remains attached to it while the ribosome decodes a few subsequent codons. This leads to asymmetric progressive elongation of ribosome footprints at the 3′-end. It is likely that internal SD sequences induce a pause not on a single, but on several adjacent codons. This finding is important for our understanding of mRNA movement through the ribosome and also should facilitate interpretation of ribosome profiling data.

**Contact:** brave.oval.pan@gmail.com

## 1 INTRODUCTION

Translation initiation sites in bacteria usually consist of a start codon (most frequently AUG, GUG or UUG) and a purine reach region 6–10 nt upstream, termed a Shine–Dalgarno (SD) sequence (Shine and Dalgarno, 1975). The SD sequence binds to the small ribosomal subunit through complementary interactions with an anti-Shine–Dalgarno (aSD) sequence located at the 3′-end of 16S rRNA (3′-AUUCCUCCAC). The importance of SD sequences for translation initiation is well known; its presence is routinely verified in algorithms for start codon prediction in bacteria (Borodovsky and Lomsadze, 2011; Hyatt *et al.*, 2010).

Until recently, the known role of internal SD sequences was limited to its stimulatory effect on ribosomal frameshifting in bacteria (Larsen *et al.*, 1994; Weiss *et al.*, 1987, 1988). Recent single-molecule studies revealed that internal SD sites can arrest ribosome movement along mRNA (Wen *et al.*, 2008). Application of the ribosome profiling technique (Ingolia *et al.*, 2009) to the analysis of protein synthesis in bacteria demonstrated that this effect is pervasive (Li *et al.*, 2012). It was shown that ribosome density is positively correlated with the presence of strong SD sequences, and strong SD–aSD interactions cause pausing of elongating ribosomes. Under conditions of fast growth, the SD site effect on translation rates seems to be more significant than that of other known coding sequence features, such as codon bias (Li *et al.*, 2012).

**Fig. 1.**
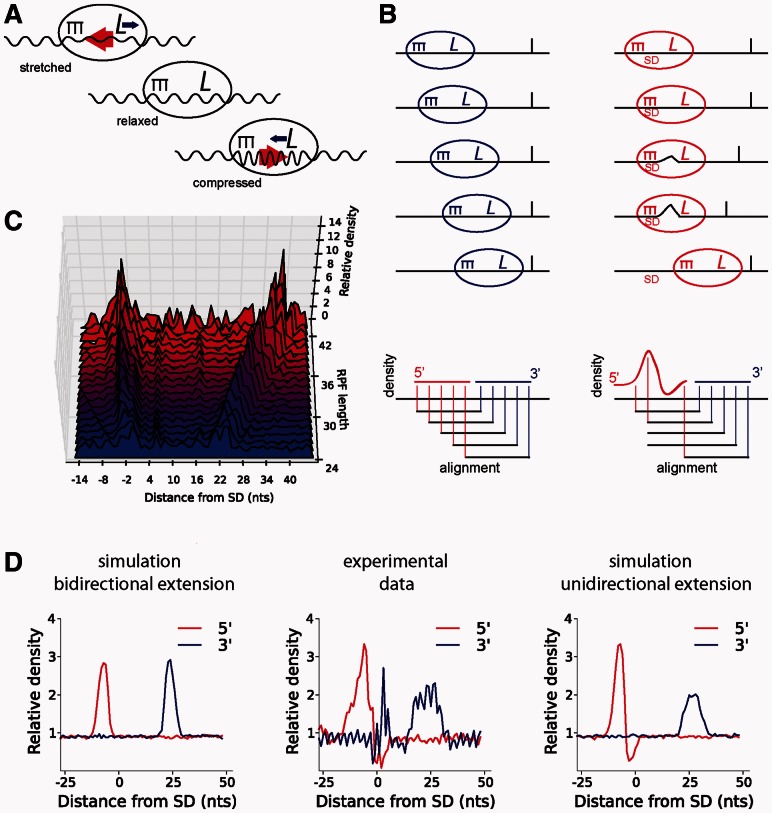
‘The caterpillar model’ of SD effect on ribosome movement. (**A**) mRNA tensions created by the distance between SD:aSD complex and the decoding center. Red arrows show the direction of the forces generated by the tensions, and blue arrows show the direction of the tRNA movement relative to the mRNA during ribosomal frameshifting. (**B**) A model of footprint generation for ribosomes moving along mRNA with (red) and without (blue) SD interactions. As ribosomes move at a constant speed, a peak of ribosome density (followed by density displacement) is expected if measured using locations of footprint 5′-ends. (**C**) Density of footprint ends (both 5′ and 3′) relative to SD site (see Section 2) for footprints of different lengths. It can be seen that the location of the 5′ peak remains unchanged, whereas the location of the 3′ peak depends on the length of footprints. (**D**) The experimental ribosome profiling data at pause sites match a model of progressive extension of footprints length. Ribosome density in the area of an SD site as estimated with the 5′- (red) and the 3′-ends (blue). The plot in the center was constructed using *E.coli* experimental data. The plot on the left is a result of a model where SD site induces a pause on a single codon, and footprint extensions are uniform at both ends. The plot on the right corresponds to a model where SD site induces a pause at three adjacent codons, resulting in asymmetrical extension of footprints relative to the P-site codons. The latter model better reflects the distribution obtained from the experimental data

However, the pausing effect of SD sequences on elongating ribosomes cannot explain all the empirical observations regarding its stimulation of ribosomal frameshifting. Indeed the effect of an SD site on frameshifting is strictly determined by its position relative to the frameshift site. For stimulation of +1 ribosomal frameshifting, an SD site needs to be located just 3 nt upstream of the P-site codon (Devaraj and Fredrick, 2010; Weiss *et al.*, 1987), whereas stimulation of −1 frameshifting requires a distance of 10–14 nt (Larsen *et al.*, 1994). In bacterial release factor 2 gene, which has a programmed frameshift site, not only the SD site but also its distance from the frameshift site is highly conserved (Baranov *et al.*, 2002). In addition to interference with a tRNA in the E-site (Marquez *et al.*, 2004), the position specificity of SD site effect on frameshifting could be due to tensions imposed on the bulk of mRNA located between the decoding center and an SD:aSD duplex. A simple analogy for such tensions is a ‘spring effect’ illustrated on Figure 1A. A short spacer is expected to produce ‘a stretched spring effect’ promoting a movement of codons in the 5′ direction relative to tRNAs (tRNAs move forward). A long spacer is expected to produce the opposite ‘compressed spring effect’. The model illustrated in Figure 1A requires SD site to be able to interact with aSD at a range of distances from the decoded codon. Such interactions would force mRNA to move through a ribosome in a caterpillar-like rippling fashion. This could occur only if ribosomes are capable of accommodating variable amount of mRNA between the SD:aSD duplex and the decoding center. In this case, ribosomes would remain bound to the same SD site while decoding several subsequent codons leading to asymmetric progressive extension of ribosome footprints as shown in Figure 1B.

In this work, we analyzed ribosome profiling data obtained for *Escherichia coli* and *Bacillus subtilis* and have shown that the length and distribution of ribosome footprints at SD site-containing mRNA regions is consistent with ‘the caterpillar model’ of progressive footprint extension.

## 2 METHODS

All ribosome profiling data are from (Li *et al.*, 2012). The footprint sequences were aligned to the organisms’ reference genomes, NC_000964.3 for *B.subtilis* and NC_000913.2 for *E.coli*. The reads were aligned allowing for two mismatches. Reads not aligning to a unique location were discarded. To ensure that footprints correspond to elongating and not initiating ribosomes, the only footprints used were those that align downstream of the first 30 nt, but upstream of the last 75 nt of coding ORFs. An SD site was defined as an octamer that is predicted to form a duplex with the aSD (5′-CACCUCCU-3′) with free energy (ΔG) <8 kcal/mol. ΔG was calculated as in (Li *et al.*, 2012) using *RNASubopt* tool from Vienna RNA package (Gruber *et al.*, 2008). We found 1206 such SD sites in 704 genes from *B.subtilis* and 1213 in 809 genes from *E.coli*. Of 21 596 339 reads aligning the genes containing strong SD sites, 1 486 956 were found to interact with SD sites (6-fold increase over expected number). For *E.coli*, 1 668 187 reads were found to interact with SD of 56 389 881 reads (3-fold increase over the expected value).

The first nucleotide of the octamer was considered as the first nucleotide of SD site irrespective of whether it forms a base pair with aSD, e.g. the C in 5′-CGGAGGUG-3′ is considered as the first nucleotide of the SD site.

**Fig. 2.**
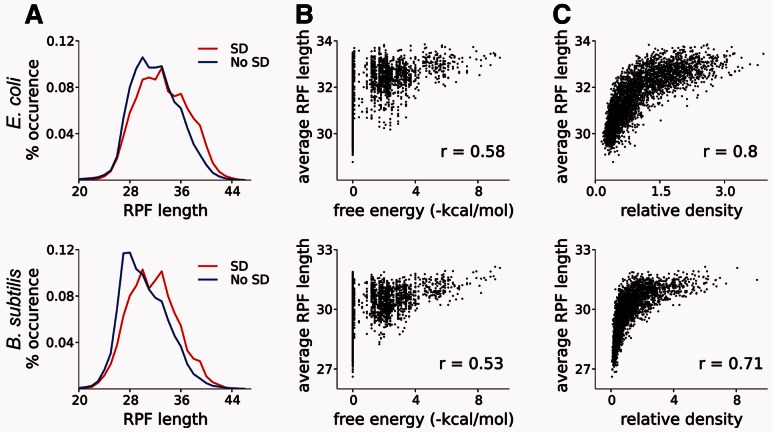
SD-containing footprints have increased length. (**A**) Length distribution of footprints (RPFs, ribosome-protected fragments) containing strong SD sequences (red) and containing no SD (blue) for *E.coli* (top) and *B.subtilis* (bottom). The difference between distributions is statistically significant; Wilcoxon rank-sum test *P* < 2.2e-16 in both cases. (**B**) Correlation between the strength of a hexamer SD:aSD duplex and the length of footprints. (**C**) Correlation between density of ribosomes on mRNA and the length of footprints

Distributions of footprint lengths (Fig. 2) were obtained for the 2122 genes of *E.coli* and 1468 in *B.subtilis* that had an average density >10 footprints per codon. To avoid unequal effects from differentially expressed genes and pause sites, the frequency of footprint occurrence of a particular length *l* was calculated as follows:
(1)
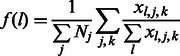

where *x_l,j,k_* is the number of footprints in SD *j* of gene *k* and *N_j_* is a number of SDs in a gene *j*. Essentially, distributions for ‘No SD’ footprints were normalized on a per gene basis, whereas distributions for ‘SD’ footprints were normalized on a per SD basis. SD-containing footprints were defined as those that have an SD site within the 3rd and 17th nt from the 5′-end of a footprint. ‘No SD’ footprints were defined as those that do not have an octamer that can form a duplex with aSD at ΔG ≤2 kcal/mol within the same region of a footprint. The significance of the difference between the two distributions in Figure 2A was estimated using the Wilcoxon rank-sum test.

The correlation analysis determined both the average normalized density and average length of fragments whose 5′-end is between 8 and 2 nt upstream for each hexanucleotide sequence (Fig. 2B and C). Similar to aforementioned data, this analysis was carried out for genes with average density >10 footprints per codon. To reduce statistical noise, the plots include data points whose averages were obtained from at least 10 values.

For the plots in Figure 1D, the *B.subtilis* footprints were mapped to the genome, and all internal SD site locations were identified using the criteria described in the first paragraph of this section. Only the regions containing SD sites (50 nt upstream and downstream of the first nucleotide in an SD site) with the average footprint density >1 per codon were used. The densities of the 5′- and 3′-ends of the footprints aggregated across all SD sites were calculated as follows:
(2)
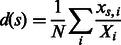

where *d(s)* is the density of footprint ends at *s* coordinate relative to SD, e.g. *s* = 1 for the first nucleotide of an SD site. *x_s,i_* is the number of footprint ends at the position *s* for SD site *i*; *X_i_* is the average number of footprints per nucleotide in the region of SD site *i*; and *N* is the total number of the SD sites used in the analysis. The plot in Figure 1C was generated in the same way, but separately for the footprints of the different lengths, i.e. in the Equation (2), both *x* and *X* values refer to the footprints of a particular length.

To simulate ribosomal profiling data *in silico*, 1000 random 1000-nt long sequences were generated. SD site positions on each sequence were determined based of free energy of the corresponding SD:aSD complex as described earlier in the text. A series of 600 footprints were generated for each sequence. Footprints were generated of a varied length (20–47 nt) with their centers corresponding to P-site codon locations. The distribution of footprint lengths was modeled based on an empirical distribution (Fig. 2A) of footprint lengths obtained from the experimental data. P-site codon locations of ribosomes were chosen randomly with equal probability *P_i_* = *P* for rapidly translated codons at coordinates *i* and *P_x_* = 9*P* for a slowly translated codons at coordinates *x*. The slowly translated codons were defined as those whose coordinates are located 11 nt downstream of an SD. To model SD site-dependent extension of footprint lengths, footprints were generated under a similar model with two alterations. First, to distribute the pause over three adjacent codons, the probability of P-site codon locations were set at *P_x_* = *P_x_*_+__3_ = *P_x_*_+__6_ = 3*P.* Second, to model asymmetric extension, footprint lengths were extended at their 5′-end for P-site codon locations at *x* + 1 to *x* + 6 by the distance between the P-site and *x*.

## 3 RESULTS AND DISCUSSION

‘The caterpillar model’ of SD-mediated footprint elongation is shown in Figure 1B. The tight association of an SD:aSD duplex could make disruption of SD:aSD duplex more energetically expensive than incorporation and folding of extra mRNA into the cavity between ribosome subunits, thus allowing for increased spacer distance between SD site and the codon in the P-site. In this scenario, footprints containing internal SD sequences in close proximity to their 5′-ends would be expected to be longer on average than footprints lacking these sequences (Fig. 1B). To ascertain whether this is the case, we compared the length of ribosome footprints obtained by (Li *et al.*, 2012) at locations with strong internal SD sites with those that found at non-SD sites (Fig. 2), see Section 2. The distribution of footprints derived from sequences not containing an SD site is skewed to the right. On average, the length of ribosome footprints without an SD site is close to the lower boundary of the distribution. By contrast, the distribution of SD-containing footprints is shifted toward longer footprints and seems more symmetric, suggesting that on average SD-containing footprints are longer, as expected from the model in Figure 1. Moreover, there is a correlation between footprint length and strength of the SD:aSD complex (Fig. 2B). The correlation is not strong (*r* = 0.58 for *B.subtilis* and *r* = 0.53 for *E.coli)* because of the large length variance of footprints not containing SD sequences (Fig. 1). However, we observed a consistent trend toward an increase in the average footprint length at stronger SD sites. If ribosome pausing and footprint elongation are both the effects of SD sequences, then a correlation between ribosome density and the length of footprints should exist. Indeed a strong correlation is observed (Fig. 2B), *r* = 0.80 for *B.subtilis* and *r* = 0.71 for *E.coli*. The correlation is nearly as strong as the correlation observed between ribosome density and the strength of SD:aSD duplex, *r* = 0.86 (Li *et al.*, 2012) and is stronger than correlation between strength of SD:aSD and the length of ribosome footprints. This raises a question regarding existence of an additional link between the length of footprints and ribosome pausing.

To investigate directionality of footprint extensions, we analyzed aggregated density of both 3′ and 5′ footprint ends relative to SD site position for the footprints of individual lengths. Figure 1C shows these distributions for footprints obtained from ribosome profiling experiments in *B.subtilis* footprints. Two distinct peaks can be observed for each footprint length, the left peak contains predominantly 5′–ends, whereas the right peak contains predominantly 3′-ends. It is clear that irrespective of the footprint length, 5′-end locations remain unchanged. At the same time, 3′-end locations shift downstream as the length of footprints increases. Therefore, SD site containing footprints are extended unidirectionaly at the 3′-ends as expected from the model in Figure 1.

To verify this observation, we designed two *in silico* models of ribosome pauses (see Section 2). The first model assumes that footprints are equally variable at both sides of a P-site codon with SD site inducing a pause at a single codon. The second model assumes that footprints at SD sites could be extended at their 3′-ends, allowing slow translation for three codons. Under the first model, similar distributions of the density would be observed for both 5′ and 3′ footprint ends with their means separated by the average length of footprints (Fig. 1D, left). In the second model, a dip can be seen shortly downstream from the 5′-end peak; also the 3′-end peak is wider than the 5′-end peak (Fig. 1D, right). The distribution of experimentally obtained footprints matches the behavior of the second model more closely (Fig. 1D, center).

Since the development of the ribosome profiling technique (Ingolia *et al.*, 2009), it has become commonly accepted that ribosome density is a direct measure of translation kinetics, e.g. a high density of ribosome footprints indicates slowly decoded mRNA locations (Ingolia *et al.*, 2011; Stadler and Fire, 2011). However, as we demonstrate here, the footprint extensions are sequence dependent and are not equivalent at the 5′- and 3′-ends. This implies that a precise position of the A-site codon in a footprint could also be variable in a sequence-dependent manner. It is possible that the length of footprints in eukaryotes may also vary because of potential interactions between mRNA and components of the ribosome. However, if they can happen, it is unlikely that such variations in eukaryotes are as pervasive as in bacteria as evidenced from the sharp triplet periodicity obtained in eukaryotic systems (Guo *et al.*, 2010; Ingolia *et al.*, 2009). The strength of triplet periodicity obtained from alignments of footprint 5′-ends is sufficient to permit detection of translated frames and switches between them (Michel *et al.*, 2012). This suggests that unlike in bacteria, the distance between the A-site and the 5′-end of eukaryotic ribosome footprints is predominantly uniform. However, if site-specific footprint length variations occur in eukaryotes on specific sequences, it would affect ribosome profiles of individual mRNAs. Therefore, it is important to take this possibility into account during interpretation of ribosome profiling data for individual mRNAs.

*Funding*: Wellcome Trust (094423 to P.V.B.); Helen Hay Whitney Foundation Fellowship (G.W.L.); Howard Hughes Medical Institute (to J.S.W.); Science Foundation Ireland (08/IN.1/B1889 to J.F.A.).

*Conflict of Interest*: none declared.
